# Supplementary figures and images for: More precise method of low-density lipoprotein cholesterol estimation for tobacco and electronic cigarette smokers: A cross-sectional study
Source: PLoS One. 2024 Sep 20;19(9):e0309002. doi: 10.1371/journal.pone.0309002 (PMC11414970; doi:10.1371/journal.pone.0309002)

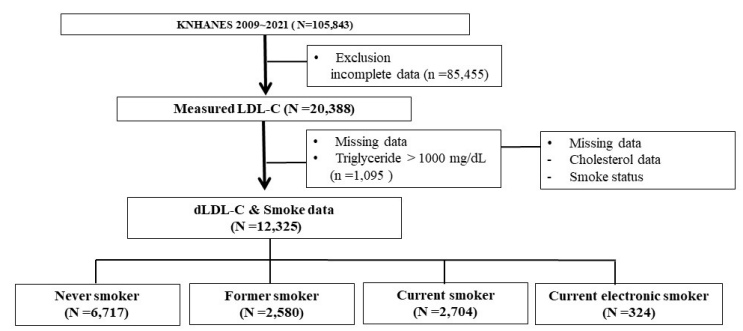


**S1 Fig**

Supplement: S1 Fig — KNHANES, Korea National Health and Nutrition Examination Survey; LDL-C, low-density lipoprotein cholesterol; dLDL-C, direct low-density lipoprotein cholesterol. (DOCX) [file pone.0309002.s001.docx]

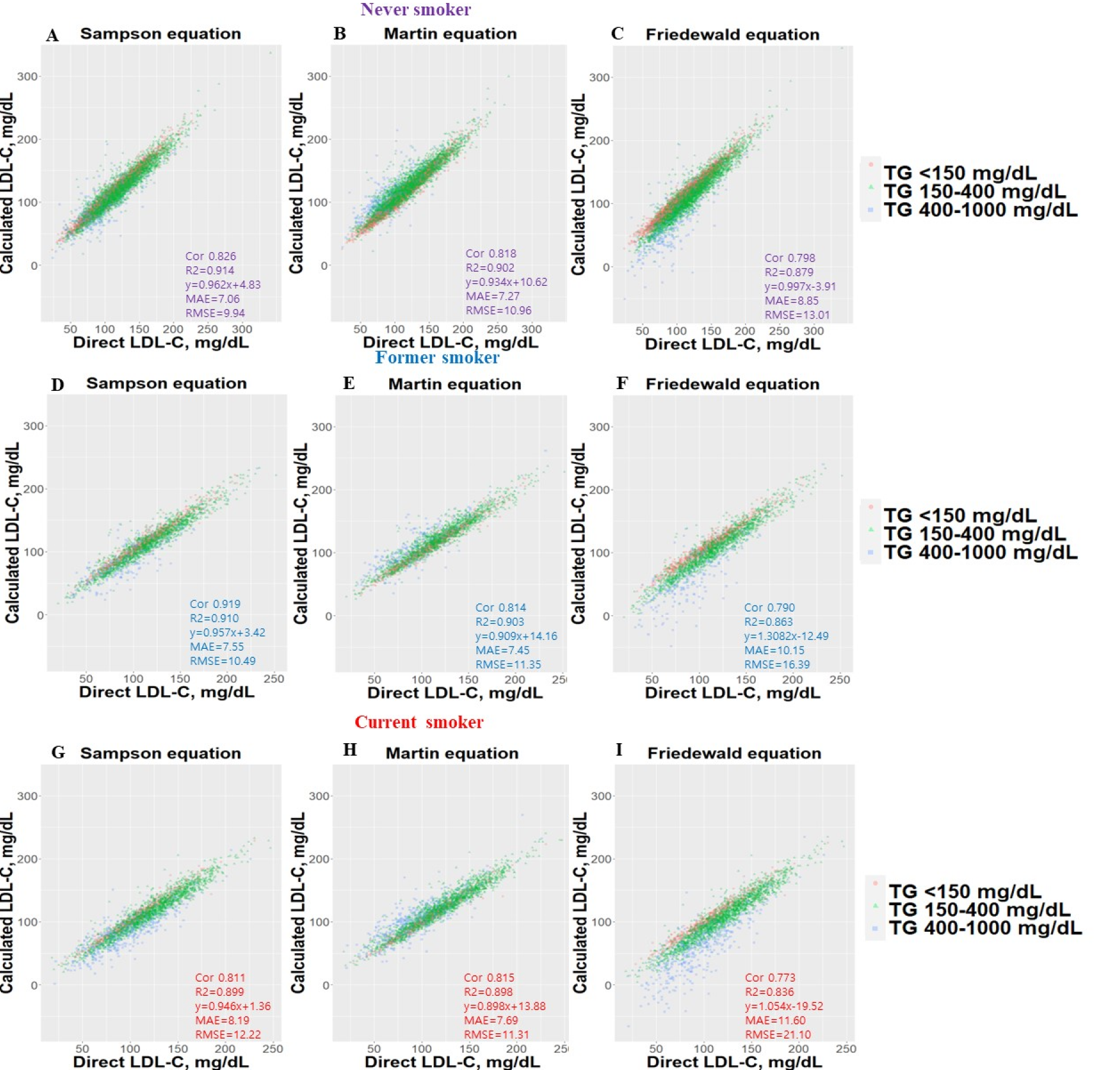


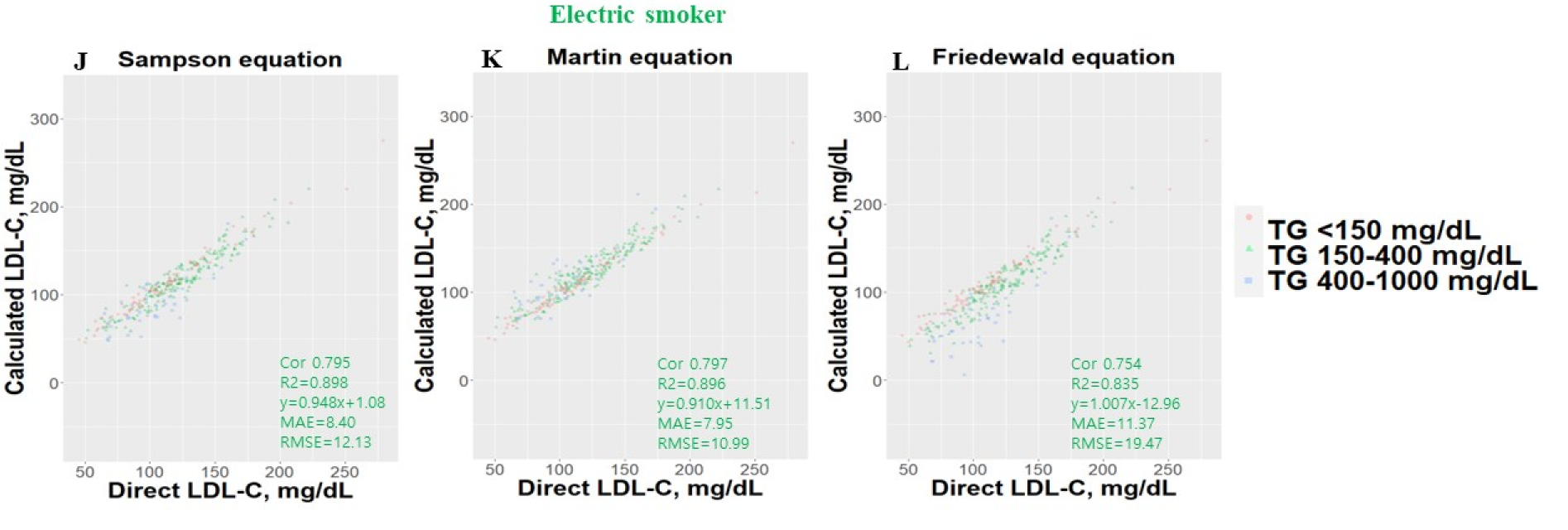


**S2 Fig**

Supplement: S2 Fig — A. The Sampson equation vs. direct LDL-C for never smokers. B. The Martin equation vs. direct LDL-C for never smokers. C. The Friedewald equation vs. direct LDL-C for never smokers. D. The Sampson equation vs. direct LDL-C for former smokers. E. The Martin equation vs. direct LDL-C for former smokers. F. The Friedewald equation vs. direct LDL-C for former smokers. G. The Sampson equation vs. direct LDL-C for current smokers. H. The Martin equation vs. direct LDL-C for current smokers. I. The Friedewald equation vs. direct LDL-C for current smokers. J. The Sampson equation vs. direct LDL-C for electronic cigarette smokers. K. The Martin equation vs. direct LDL-C for electronic cigarette smokers. L. The Friedewald equation vs. direct LDL-C for electronic cigarette smokers. Cor, correlation coefficient; MAE, mean absolute error; R2, correlation coefficient; RMSE, root mean square error; TG, triglyceride. The dots indicate the individual samples colored according to TG level. The color scale indicates individuals. SI conversion factors: To convert cholesterol to mmol/L, values were multiplied by 0.0259. (DOCX) [file pone.0309002.s002.docx]

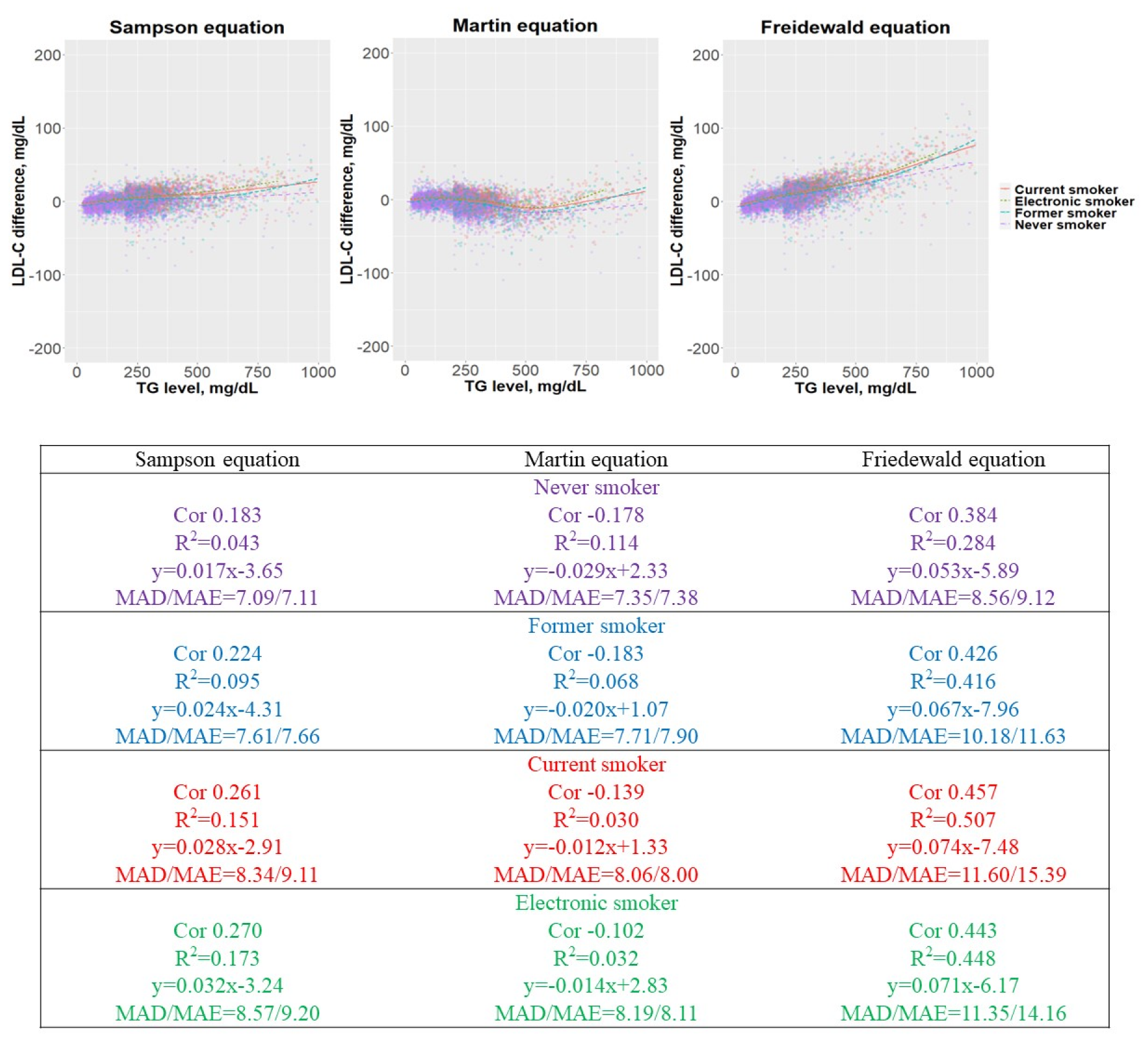


**S3 Fig**

Supplement: S3 Fig — (A) MAD of estimated LDL-C vs. dLDL-C for the never smoker group (TG levels <1000 mg/dL). (B) MAD of estimated LDL-C vs. dLDL-C for the never smoker group (TG levels <150 mg/dL). (C) MAD of estimated LDL-C vs. dLDL-C for the former smoker group (TG levels <1000 mg/dL). (D) MAD of estimated LDL-C vs. dLDL-C for the former smoker group (TG levels <150 mg/dL). (E) MAD of estimated LDL-C vs. dLDL-C for the current smoker group (TG levels <150 mg/dL). (F) MAD of estimated LDL-C vs. dLDL-C for the current smoker group (TG levels <150 mg/dL). (G) MAD of estimated LDL-C vs. dLDL-C for the EC smoker group (TG levels <150 mg/dL). (H) MAD of estimated LDL-C vs. dLDL-C for the EC smoker group (TG levels <150 mg/dL). HDL, high-density lipoprotein; TG, triglyceride; EC, electronic cigarette. Sampson equation (blue), Martin equation (red), and Friedewald equation (green). (DOCX) [file pone.0309002.s003.docx]

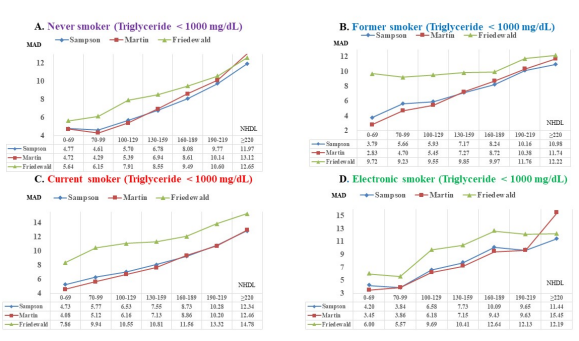


**S4 Fig**

Supplement: S4 Fig — (A) MAD of estimated LDL-C vs. dLDL-C for the never smoker group (TG levels <1000 mg/dL). (B) MAD of estimated LDL-C vs. dLDL-C for the former smoker group (TG levels <1000 mg/dL). (C) MAD of estimated LDL-C vs. dLDL-C for the current smoker group (TG levels <150 mg/dL). (D) MAD of estimated LDL-C vs. dLDL-C for the EC smoker group (TG levels <150 mg/dL. NHDL, non-high-density lipoprotein; TG, triglyceride; EC, electronic cigarette. Sampson equation (blue), Martin equation (red), and Friedewald equation (green). (DOCX) [file pone.0309002.s004.docx]

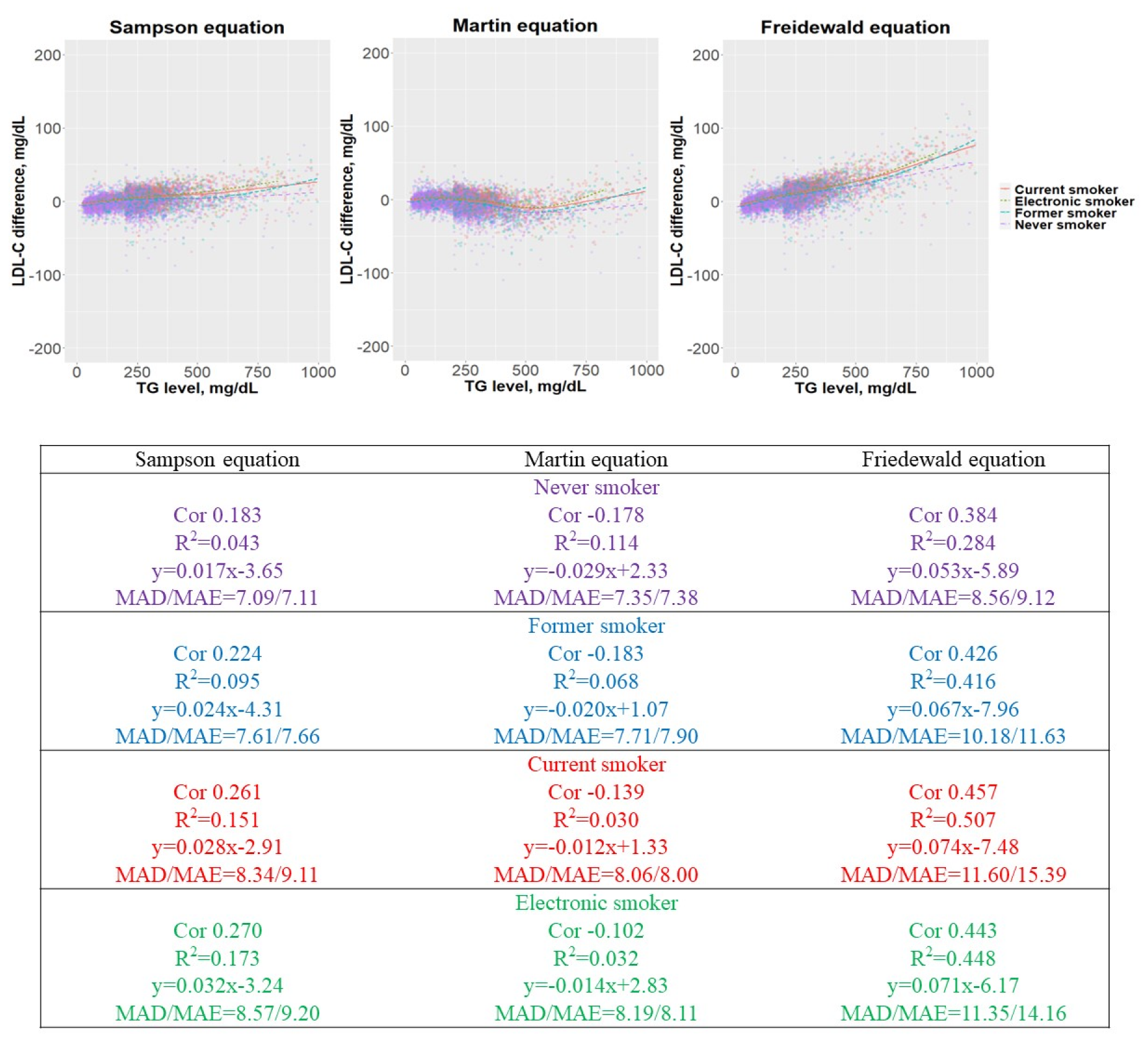


**S5 Fig**

Supplement: S5 Fig — Differences between the estimates obtained by the Sampson equation (A), Martin equation (B), and Friedewald equation (C) and direct LDL-C (dLDL-C), stratified by triglyceride (TG) level. Residual error was calculated by the difference between direct LDL-C (dLDL-C) and the values obtained using the equations. The dots indicate the individual samples, colored according to smoking status. The color scale indicates individuals. The solid line indicates the trend by the local regression method. Cor, correlation coefficient; MAD, mean absolute deviation; MAE, mean absolute error; R2 indicates correlation coefficient. (DOCX) [file pone.0309002.s005.docx]
