# Supplementary material for: More precise method of low-density lipoprotein cholesterol estimation for tobacco and electronic cigarette smokers: A cross-sectional study
Source: PLoS One. 2024 Sep 20;19(9):e0309002. doi: 10.1371/journal.pone.0309002 (PMC11414970; doi:10.1371/journal.pone.0309002)
Supplement: S1 Table — (DOCX) [file pone.0309002.s006.docx]

S1 Table. Characteristics of the study population with triglyceride levels of ≥400 mg/dL and <1000 mg/dL

| **Smoke status** | **Never** | **Former** | **Current** | **Electronic** | **P-value** |
| --- | --- | --- | --- | --- | --- |
|  | **(N=324)** | **(N=249)** | **(N=420)** | **(N=40)** |  |
| **Age, years** | 51.0 [39.0;61.5] | 53.0 [45.0;61.0] | 46.0 [40.0;54.0] | 40.5 [37.0;45.5] | **<0.001** |
| **Male, %** | 121 (37.3%) | 235 (94.4%) | 378 (90.0%) | 39 (97.5%) | **<0.001** |
| **Body mass index, kg/m^2^** | 25.8 [23.9;27.8] | 25.5 [23.8;27.5] | 25.4 [23.4;27.7] | 25.7 [23.9;28.7] | 0.391 |
| **Laboratory data** |  |  |  |  |  |
| **Fasting glucose, mg/dL** | 102.5 [95.0;122.0] | 106.0 [97.0;127.0] | 103.0 [95.0;121.0] | 97.0 [92.0;109.5] | **0.026** |
| **Blood urea nitrogen, mg/dL** | 14.0 [12.0;17.0] | 16.0 [13.0;19.0] | 14.0 [12.0;17.0] | 14.0 [11.0;17.0] | **<0.001** |
| **Creatinine, mg/dL** | 0.8 [ 0.6; 0.9] | 0.9 [ 0.8; 1.0] | 0.9 [ 0.8; 1.0] | 0.9 [ 0.8; 0.9] | **<0.001** |
| **GFR_EPI** | 98.7 [83.4;111.5] | 95.2 [83.7;102.5] | 100.5 [91.4;110.0] | 102.1 [94.5;113.0] | **<0.001** |
| **Cholesterol** |  |  |  |  |  |
| **Total cholesterol, mg/dL** | 213.0 [189.0;242.0] | 217.0 [186.0;244.0] | 215.0 [190.0;240.0] | 212.0 [195.0;243.5] | 0.962 |
| **Triglyceride, mg/dL** | 490.5 [435.0;586.0] | 491.0 [433.0;607.0] | 538.0 [451.0;669.0] | 506.0 [459.5;618.0] | **<0.001** |
| **HDL-C, mg/dL** | 37.0 [32.0;42.0] | 37.3 [31.2;43.9] | 38.0 [33.0;43.0] | 37.5 [31.7;42.8] | 0.674 |
| **Direct LDL-C, mg/dL** | 92.5 [74.0;115.0] | 94.0 [72.0;116.0] | 94.0 [75.5;118.0] | 104.0 [85.0;119.0] | 0.200 |
| **Non-HDL-C, mg/dL** | 177.0 [153.4;205.5] | 176.0 [147.0;203.0] | 174.0 [151.4;201.0] | 176.3 [156.2;202.9] | 0.818 |
| **Sampson, mg/dL** | 89.5 [69.8;109.1] | 83.6 [65.9;111.2] | 83.7 [63.5;103.1] | 88.5 [73.7;106.9] | 0.063 |
| **Martin, mg/dL** | 109.6 [89.5;129.7] | 104.0 [85.6;131.5] | 103.8 [83.0;124.3] | 108.7 [93.5;129.3] | 0.075 |
| **Friedewald, mg/dL** | 70.7 [44.6;96.3] | 64.8 [39.9;98.4] | 63.2 [34.1;87.8] | 70.1 [46.2;91.7] | **0.025** |
| **Positive absolute value Martin, mg/dL** | 11.5 [ 4.5;19.9] | 10.0 [ 5.4;19.8] | 14.5 [ 6.6;24.6] | 17.4 [10.2;26.0] | **0.001** |
| **Positive absolute value Sampson, mg/dL** | 17.7 [ 8.5;27.0] | 15.4 [ 8.5;25.3] | 12.0 [ 5.8;20.4] | 11.9 [ 4.8;21.0] | **<0.001** |
| **Positive absolute value Friedewald, mg/dL** | 23.0 [13.2;36.1] | 25.4 [13.4;40.4] | 33.1 [19.9;46.2] | 32.9 [18.6;48.8] | **<0.001** |

| **Smoke status**  Adjusted p-values (Benjamini–Hochberg method) | **Never** | **Never** | **Never** | **Former** | **Former** | **Current** |
| --- | --- | --- | --- | --- | --- | --- |
|  | **Former** | **Current** | **Electronic** | **Current** | **Electronic** | **Electronic** |
| **Age, years** | **0.037** | **0.001** | **<0.001** | **<0.001** | **<0.001** | **0.001** |
| **Male, %** | **<0.001** | **<0.001** | **<0.001** | 0.101 | 0.658 | 0.244 |
| **Body mass index, kg/m^2^** | 0.602 | 0.602 | 0.768 | 0.602 | 0.602 | 0.602 |
| **Laboratory data** |  |  |  |  |  |  |
| **Fasting glucose, mg/dL** | 0.088 | 0.600 | 0.108 | 0.137 | **0.030** | 0.088 |
| **Blood urea nitrogen, mg/dL** | **<0.001** | 0.691 | 0.691 | **<0.001** | **0.016** | 0.691 |
| **Creatinine, mg/dL** | **<0.001** | **<0.001** | **<0.001** | **0.002** | 0.350 | 0.523 |
| **GFR_EPI** | **0.004** | 0.224 | 0.168 | **<0.001** | **<0.001** | 0.224 |
| **Cholesterol** |  |  |  |  |  |  |
| **Total cholesterol, mg/dL** | 0.998 | 0.998 | 0.998 | 0.998 | 0.998 | 0.998 |
| **Triglyceride, mg/dL** | 0.808 | **<0.001** | 0.492 | **0.006** | 0.530 | 0.492 |
| **HDL-C, mg/dL** | 0.971 | 0.971 | 0.971 | 0.971 | 0.971 | 0.971 |
| **Direct LDL-C, mg/dL** | 0.983 | 0.642 | 0.144 | 0.642 | 0.144 | 0.158 |
| **Non-HDL-C, mg/dL** | 0.978 | 0.978 | 0.978 | 0.978 | 0.978 | 0.978 |
| **Sampson, mg/dL** | 0.416 | 0.054 | 0.977 | 0.416 | 0.690 | 0.416 |
| **Martin, mg/dL** | 0.387 | 0.066 | 0.917 | 0.387 | 0.661 | 0.387 |
| **Friedewald, mg/dL** | 0.366 | **0.018** | 0.902 | 0.366 | 0.707 | 0.366 |
| **Positive absolute value Martin, mg/dL** | 0.877 | **0.006** | 0.030 | **0.006** | 0.024 | 0.364 |
| **Positive absolute value Sampson, mg/dL** | 0.217 | **<0.001** | 0.080 | **0.003** | 0.204 | 0.993 |
| **Positive absolute value Friedewald, mg/dL** | 0.181 | **<0.001** | 0.056 | **<0.001** | 0.181 | 0.740 |

HDL-C, high-density lipoprotein cholesterol; LDL-C, low-density lipoprotein cholesterol.

The positive absolute value is direct LDL-C minus calculated by lipid equations (Friedewald, Sampson, and Martin). SI conversion factors: To convert cholesterol to mmol/L, values are multiplied by 0.0259.
