# Supplementary material for: More precise method of low-density lipoprotein cholesterol estimation for tobacco and electronic cigarette smokers: A cross-sectional study
Source: PLoS One. 2024 Sep 20;19(9):e0309002. doi: 10.1371/journal.pone.0309002 (PMC11414970; doi:10.1371/journal.pone.0309002)
Supplement: S2 Table — (DOCX) [file pone.0309002.s007.docx]

S2 Table. Characteristics of the study population with triglyceride levels of ≥150 mg/dL and <400 mg/dL

| **Smoke status** | **Never** | **Former** | **Current** | **Electronic** | **P-value** |
| --- | --- | --- | --- | --- | --- |
|  | **(N=3434)** | **(N=1660)** | **(N=1839)** | **(N=205)** |  |
| **Age, years** | 55.0 [41.0;66.0] | 56.0 [45.0;66.0] | 48.0 [39.0;58.0] | 41.0 [33.0;49.0] | **<0.001** |
| **Male, %** | 916 (26.7%) | 1531 (92.2%) | 1651 (89.8%) | 188 (91.7%) | **<0.001** |
| **Body mass index, kg/m^2^** | 25.2 [23.0;27.5] | 25.4 [23.6;27.6] | 25.2 [23.2;27.4] | 25.5 [23.6;28.1] | **0.021** |
| **Laboratory data** |  |  |  |  |  |
| **Fasting glucose, mg/dL** | 99.0 [92.0;111.0] | 103.0 [95.0;116.0] | 100.0 [93.0;113.0] | 99.0 [92.0;109.0] | **<0.001** |
| **Blood urea nitrogen, mg/dL** | 14.0 [11.0;17.0] | 15.0 [12.0;18.0] | 14.0 [11.0;16.0] | 13.0 [11.0;16.0] | **<0.001** |
| **Creatinine, mg/dL** | 0.7 [ 0.6; 0.9] | 0.9 [ 0.8; 1.0] | 0.9 [ 0.8; 1.0] | 0.9 [ 0.8; 1.0] | **<0.001** |
| **GFR_EPI** | 97.5 [82.6;110.7] | 90.0 [79.5;100.1] | 96.7 [85.3;106.3] | 100.0 [90.8;109.1] | **<0.001** |
| **Cholesterol** |  |  |  |  |  |
| **Total cholesterol, mg/dL** | 203.0 [180.0;231.0] | 201.0 [176.0;225.5] | 203.0 [178.0;228.5] | 204.0 [182.0;231.0] | **0.009** |
| **Triglyceride, mg/dL** | 233.0 [208.0;277.0] | 244.0 [214.0;290.0] | 247.0 [216.0;296.0] | 257.0 [217.0;305.0] | **<0.001** |
| **HDL-C, mg/dL** | 42.3 [37.2;48.7] | 42.0 [37.0;48.0] | 41.3 [36.0;48.0] | 41.0 [36.5;47.3] | **<0.001** |
| **Direct LDL-C, mg/dL** | 118.0 [96.0;142.0] | 115.0 [93.0;139.0] | 120.0 [97.0;143.5] | 122.0 [102.0;144.0] | **0.001** |
| **Non-HDL-C, mg/dL** | 159.7 [137.0;185.7] | 157.7 [134.7;181.0] | 160.0 [135.0;184.7] | 162.7 [142.0;187.5] | **0.020** |
| **Sampson, mg/dL** | 116.6 [95.9;140.7] | 113.5 [91.3;135.9] | 114.2 [92.5;138.4] | 115.3 [98.8;137.6] | **<0.001** |
| **Martin, mg/dL** | 122.0 [101.4;145.6] | 118.9 [98.4;140.8] | 121.0 [98.7;143.9] | 121.1 [105.9;143.9] | **0.002** |
| **Friedewald, mg/dL** | 110.6 [88.1;135.9] | 107.2 [82.6;130.8] | 107.2 [84.6;133.3] | 107.7 [89.5;131.2] | **<0.001** |
| **Positive absolute value Martin, mg/dL** | 6.3 [ 2.9;11.3] | 6.3 [ 3.2;10.7] | 7.4 [ 3.4;12.4] | 8.0 [ 3.6;13.8] | **<0.001** |
| **Positive absolute value Sampson, mg/dL** | 6.5 [ 3.0;11.6] | 6.0 [ 2.6;10.5] | 5.8 [ 2.8;10.2] | 6.9 [ 3.1;11.5] | **<0.001** |
| **Positive absolute value Friedewald, mg/dL** | 9.4 [ 4.8;15.2] | 10.5 [ 5.3;16.4] | 12.4 [ 6.8;18.6] | 12.0 [ 6.1;19.3] | **<0.001** |

| **Smoke status**  Adjusted p-values (Benjamini–Hochberg method) | **Never** | **Never** | **Never** | **Former** | **Former** | **Current** |
| --- | --- | --- | --- | --- | --- | --- |
|  | **Former** | **Current** | **Electronic** | **Current** | **Electronic** | **Electronic** |
| **Age, years** | 0.001 | **<0.001** | **<0.001** | **<0.001** | **<0.001** | **<0.001** |
| **Male, %** | **<0.001** | **<0.001** | **<0.001** | 0.021 | 0.901 | 0.5436 |
| **Body mass index, kg/m^2^** | **0.048** | 0.888 | 0.174 | 0.066 | 0.634 | 0.174 |
| **Laboratory data** |  |  |  |  |  |  |
| **Fasting glucose, mg/dL** | **<0.001** | **0.020** | 0.413 | **<0.001** | **<0.001** | 0.084 |
| **Blood urea nitrogen, mg/dL** | **<0.001** | 0.517 | 0.396 | **<0.001** | **<0.001** | 0.478 |
| **Creatinine, mg/dL** | **<0.001** | **<0.001** | **<0.001** | **<0.001** | **0.014** | 0.853 |
| **GFR_EPI** | **<0.001** | **0.018** | 0.035 | **<0.001** | **<0.001** | **<0.001** |
| **Cholesterol** |  |  |  |  |  |  |
| **Total cholesterol, mg/dL** | **0.006** | 0.201 | 0.709 | 0.201 | 0.201 | 0.378 |
| **Triglyceride, mg/dL** | **<0.001** | **<0.001** | **<0.001** | **0.008** | **0.030** | 0.338 |
| **HDL-C, mg/dL** | **0.042** | **<0.001** | 0.219 | 0.219 | 0.719 | 0.719 |
| **Direct LDL-C, mg/dL** | **0.012** | 0.329 | 0.195 | **0.006** | **0.018** | 0.329 |
| **Non-HDL-C, mg/dL** | **0.024** | 0.547 | 0.518 | 0.100 | 0.100 | 0.500 |
| **Sampson, mg/dL** | **<0.001** | **0.009** | 0.681 | 0.378 | 0.378 | 0.538 |
| **Martin, mg/dL** | **<0.001** | 0.090 | 0.934 | 0.224 | 0.224 | 0.541 |
| **Friedewald, mg/dL** | **<0.001** | **0.003** | 0.541 | 0.461 | 0.461 | 0.541 |
| **Positive absolute value Martin, mg/dL** | 0.605 | **<0.001** | **0.003** | **<0.001** | **0.002** | 0.149 |
| **Positive absolute value Sampson, mg/dL** | 0.003 | **<0.001** | 0.968 | 0.581 | 0.248 | 0.140 |
| **Positive absolute value Friedewald, mg/dL** | **<0.001** | **<0.001** | **<0.001** | **<0.001** | **0.004** | 0.810 |

HDL-C, high-density lipoprotein cholesterol; LDL-C, low-density lipoprotein cholesterol.

The positive absolute value is direct LDL-C minus calculated by lipid equations (Friedewald, Sampson, and Martin). SI conversion factors: To convert cholesterol to mmol/L, values are multiplied by 0.0259.
