# Supplementary material for: More precise method of low-density lipoprotein cholesterol estimation for tobacco and electronic cigarette smokers: A cross-sectional study
Source: PLoS One. 2024 Sep 20;19(9):e0309002. doi: 10.1371/journal.pone.0309002 (PMC11414970; doi:10.1371/journal.pone.0309002)
Supplement: S3 Table — (DOCX) [file pone.0309002.s008.docx]

S3 Table. Characteristics of the study population with triglyceride levels of <150 mg/dL

| **Smoke status** | **Never** | **Former** | **Current** | **Electronic** | **P-value** |
| --- | --- | --- | --- | --- | --- |
|  | **(N=2959)** | **(N=671)** | **(N=445)** | **(N=79)** |  |
| **Age, years** | 43.0 [23.0;59.0] | 59.0 [46.0;70.0] | 48.0 [35.0;60.0] | 33.0 [24.0;44.5] | **<0.001** |
| **Male, %** | 700 (23.7%) | 572 (85.2%) | 367 (82.5%) | 62 (78.5%) | **<0.001** |
| **Body mass index, kg/m^2^** | 22.5 [20.5;24.8] | 23.9 [22.0;25.9] | 23.2 [20.8;25.3] | 23.2 [21.1;26.0] | **<0.001** |
| **Laboratory data** |  |  |  |  |  |
| **Fasting glucose, mg/dL** | 92.0 [87.0;99.0] | 97.0 [90.0;106.5] | 94.0 [88.0;101.0] | 93.0 [89.0;101.5] | **<0.001** |
| **Blood urea nitrogen, mg/dL** | 13.0 [11.0;16.0] | 15.0 [13.0;19.0] | 14.0 [12.0;16.0] | 13.0 [11.0;15.0] | **<0.001** |
| **Creatinine, mg/dL** | 0.7 [ 0.6; 0.8] | 0.9 [ 0.8; 1.0] | 0.9 [ 0.8; 1.0] | 0.9 [ 0.8; 1.0] | **<0.001** |
| **GFR_EPI** | 103.7 [88.3;121.1] | 87.5 [75.0;97.6] | 96.5 [85.4;107.3] | 103.7 [94.2;112.9] | **<0.001** |
| **Cholesterol** |  |  |  |  |  |
| **Total cholesterol, mg/dL** | 179.0 [157.0;204.0] | 181.0 [158.0;203.0] | 177.0 [155.0;199.0] | 178.0 [157.0;198.5] | 0.337 |
| **Triglyceride, mg/dL** | 82.0 [60.0;108.0] | 94.0 [71.0;117.5] | 96.0 [72.0;121.0] | 93.0 [73.0;115.5] | **<0.001** |
| **HDL-C, mg/dL** | 53.4 [46.3;62.5] | 49.3 [42.3;58.4] | 50.4 [43.3;59.4] | 50.4 [43.8;56.4] | **<0.001** |
| **Direct LDL-C, mg/dL** | 107.0 [87.0;128.5] | 110.0 [88.5;130.0] | 106.0 [87.0;126.0] | 107.0 [87.5;131.5] | 0.386 |
| **Non-HDL-C, mg/dL** | 124.6 [103.5;147.8] | 129.6 [107.1;151.6] | 124.7 [104.5;145.6] | 126.5 [103.3;149.0] | **0.045** |
| **Sampson, mg/dL** | 109.4 [88.7;131.3] | 112.0 [91.4;133.2] | 106.7 [87.4;129.1] | 106.6 [88.4;133.8] | 0.137 |
| **Martin, mg/dL** | 107.0 [87.0;128.4] | 110.2 [89.8;130.9] | 105.7 [85.9;126.8] | 105.5 [86.3;131.1] | 0.122 |
| **Friedewald, mg/dL** | 108.1 [87.9;129.5] | 109.9 [90.5;131.2] | 105.1 [86.2;127.1] | 106.3 [87.1;131.4] | 0.126 |
| **Positive absolute value Martin, mg/dL** | 4.2 [ 1.8; 7.4] | 3.9 [ 1.8; 7.1] | 3.6 [ 1.5; 6.3] | 3.5 [ 1.6; 6.7] | **0.031** |
| **Positive absolute value Sampson, mg/dL** | 3.5 [ 1.8; 6.3] | 3.4 [ 1.7; 6.2] | 3.5 [ 1.5; 5.5] | 3.7 [ 2.1; 7.4] | 0.243 |
| **Positive absolute value Friedewald, mg/dL** | 4.1 [ 2.0; 7.1] | 3.8 [ 1.8; 7.1] | 3.9 [ 2.2; 6.2] | 4.2 [ 2.1; 7.6] | 0.476 |

| **Smoke status**  Adjusted p-values (Benjamini–Hochberg method) | **Never** | **Never** | **Never** | **Former** | **Former** | **Current** |
| --- | --- | --- | --- | --- | --- | --- |
|  | **Former** | **Current** | **Electronic** | **Current** | **Electronic** | **Electronic** |
| **Age, years** | **<0.001** | **<0.001** | 0.026 | **<0.001** | **<0.001** | **<0.001** |
| **Male, %** | **<0.001** | **<0.001** | **<0.001** | 0.2964 | 0.2385 | 0.49 |
| **Body mass index, kg/m^2^** | **<0.001** | **0.008** | 0.074 | **<0.001** | 0.253 | 0.454 |
| **Laboratory data** |  |  |  |  |  |  |
| **Fasting glucose, mg/dL** | **<0.001** | **0.021** | 0.302 | **<0.001** | **0.012** | 0.959 |
| **Blood urea nitrogen, mg/dL** | **<0.001** | **<0.001** | 0.475 | **<0.001** | **<0.001** | **0.014** |
| **Creatinine, mg/dL** | **<0.001** | **<0.001** | **<0.001** | **<0.001** | **0.024** | 0.845 |
| **GFR_EPI** | **<0.001** | **<0.001** | 0.700 | **<0.001** | **<0.001** | **<0.001** |
| **Cholesterol** |  |  |  |  |  |  |
| **Total cholesterol, mg/dL** | 0.904 | 0.369 | 0.904 | 0.369 | 0.904 | 0.904 |
| **Triglyceride, mg/dL** | **<0.001** | **<0.001** | **0.004** | 0.611 | 0.857 | 0.857 |
| **HDL-C, mg/dL** | **<0.001** | **<0.001** | **0.044** | 0.410 | 0.730 | 0.882 |
| **Direct LDL-C, mg/dL** | 0.555 | 0.779 | 0.863 | 0.546 | 0.906 | 0.779 |
| **Non-HDL-C, mg/dL** | **0.036** | 0.843 | 0.835 | 0.099 | 0.835 | 0.835 |
| **Sampson, mg/dL** | 0.297 | 0.340 | 0.886 | 0.120 | 0.678 | 0.784 |
| **Martin, mg/dL** | 0.159 | 0.548 | 0.972 | 0.126 | 0.692 | 0.797 |
| **Friedewald, mg/dL** | 0.282 | 0.282 | 0.818 | 0.096 | 0.656 | 0.784 |
| **Positive absolute value Martin, mg/dL** | 0.552 | 0.030 | 0.500 | 0.234 | 0.552 | 0.887 |
| **Positive absolute value Sampson, mg/dL** | 0.597 | 0.363 | 0.402 | 0.402 | 0.402 | 0.363 |
| **Positive absolute value Friedewald, mg/dL** | 0.600 | 0.600 | 0.805 | 0.988 | 0.600 | 0.600 |

HDL-C, high-density lipoprotein cholesterol; LDL-C, low-density lipoprotein cholesterol.

The positive absolute value is direct LDL-C minus calculated by lipid equations (Friedewald, Sampson, and Martin). SI conversion factors: To convert cholesterol to mmol/L, values were multiplied by 0.0259.
