# Supplementary material for: More precise method of low-density lipoprotein cholesterol estimation for tobacco and electronic cigarette smokers: A cross-sectional study
Source: PLoS One. 2024 Sep 20;19(9):e0309002. doi: 10.1371/journal.pone.0309002 (PMC11414970; doi:10.1371/journal.pone.0309002)
Supplement: S4 Table — (DOCX) [file pone.0309002.s009.docx]

S4 Table. Mean and median absolute deviations with 95% confidence intervals of estimated low-density lipoprotein cholesterol stratified by dLDL-C in the group with TG levels of <400 mg/dL

|  | **Sampson equation** | | **Martin equation** | | **Friedewald equation** | |
| --- | --- | --- | --- | --- | --- | --- |
|  |  |  |  | **Never smoker** |  |  |
| dLDL-C | MAD | MeAD | MAD | MeAD | MAD | MeAD |
| **<40** | 4.31 | -0.89 (-4.57 to 2.79) | 4.39 | -1.78 (-5.61 to 2.05) | 7.45 | -1.66 (-6.27 to 2.95) |
| **<70** | 6.17 | -3.34 (-4.84 to -1.83) | 6.34 | -1.85 (-3.25 to -0.46) | 8.82 | -6.35 (-8.15 to -4.56) |
| **≥70 and <100** | 6.01 | -1.19 (-1.86 to -0.51) | 6.50 | -0.74 (-1.40 to -0.08) | 7.60 | -2.37 (-3.11 to -1.63) |
| **≥100 and <130** | 6.70 | -2.52 (-3.17 to -1.87) | 6.59 | -1.48 (-2.09 to -0.88) | 7.92 | -3.18 (-3.86 to -2.51) |
| **≥130 & <160** | 6.89 | -2.52 (-3.39 to -1.66) | 6.53 | -1.85 (-2.68 to -1.03) | 7.63 | -3.00 (-3.89 to -2.11) |
| **≥160** | 8.52 | -2.45 (-5.57 to 0.68) | 7.60 | -1.04 (-4.11 to 2.04) | 8.96 | -3.19 (-6.35 to -0.03) |
|  |  |  |  | Former smoker |  |  |
| **dLDL-C** | MAD | MeAD | MAD | MeAD | MAD | MeAD |
| **<40** | 5.85 | -0.89 (-7.28 to 5.50) | 5.70 | -1.11 (-7.34 to 5.11) | 9.69 | -9.91 (-18.11 to -1.71) |
| **<70** | 6.49 | -3.56 (-7.52 to 0.41) | 6.32 | -1.04 (-4.86 to 2.78) | 9.68 | -6.02 (-10.16 to -1.88) |
| **≥70 and <100** | 6.69 | -0.30 (-1.58 to 0.98) | 6.45 | -0.74 (-2.06 to 0.58) | 9.09 | -2.58 (-4.05 to -1.11) |
| **≥100 and <130** | 6.48 | -1.41 (-2.40 to -0.42) | 6.23 | -1.33 (-2.31 to -0.36) | 8.03 | -2.62 (-3.68 to -1.55) |
| **≥130 and <160** | 7.00 | -2.08 (-3.45 to -0.70) | 6.30 | -1.85 (-3.19 to -0.52) | 7.91 | -2.90 (-4.35 to -1.46) |
| **≥160** | 8.94 | -2.97 (-7.86 to 1.93) | 8.03 | -1.48 (-6.33 to 3.36) | 9.38 | -4.01 (-8.97 to 0.96) |
|  |  |  |  | Current smoker |  |  |
| **dLDL-C** | MAD | MeAD | MAD | MeAD | MAD | MeAD |
| **<40** | 5.62 | -4.89 (-10.43 to 0.65) | 6.67 | -4.67 (-10.94 to 1.60) | 7.48 | -9.12 (-15.89 to -2.34) |
| **<70** | 6.52 | -2.15 (-6.24 to 1.94) | 7.00 | -0.37 (-4.38 to 3.64) | 9.15 | -6.07 (-10.42 to -1.73) |
| **≥70 and <100** | 6.25 | -2.52 (-3.88 to -1.16) | 5.94 | -1.63 (-2.92 to -0.34) | 8.77 | -5.63 (-7.21 to -4.06) |
| **≥100 and <130** | 6.92 | -0.89 (-1.89 to 0.11) | 6.43 | -0.89 (-1.88 to 0.10) | 8.51 | -2.38 (-3.47 to -1.28) |
| **≥130 and <160** | 7.51 | -0.74 (-2.05 to 0.56) | 6.86 | -0.89 (-2.20 to 0.43) | 8.44 | -1.66 (-3.04 to -0.28) |
| **≥160** | 8.11 | -5.04 (-7.93 to -2.15) | 7.12 | -2.82 (-5.53 to -0.10) | 8.66 | -6.18 (-9.20 to -3.16) |
|  |  |  |  | Electronic cigarette smoker |  |  |
| **dLDL-C** | MAD | MeAD | MAD | MeAD | MAD | MeAD |
| **<40** |  |  |  |  |  |  |
| **<70** | 5.83 | -5.19 (-10.04 to -0.34) | 7.22 | -7.71 (-14.54 to -0.88) | 8.10 | -6.98 (-12.89 to -1.08) |
| **≥70 and <100** | 7.54 | -1.85 (-5.70 to 1.99) | 6.81 | -1.70 (-5.50 to 2.09) | 10.23 | -7.38 (-12.20 to -2.56) |
| **≥100 and <130** | 6.75 | 1.11 (-1.34 to 3.56) | 6.71 | -0.07 (-2.62 to 2.48) | 8.24 | 0.76 (-1.75 to 3.28) |
| **≥130 and <160** | 7.56 | -2.52 (-8.00 to 2.96) | 6.95 | -1.63 (-7.08 to 3.82) | 8.73 | -3.08 (-8.62 to 2.45) |
| **≥160** | 8.30 | -11.34 (-21.01 to -1.67) | 6.85 | -9.41 (-18.95 to 0.12) | 9.09 | -13.2 (-23.31 to -3.08) |

CI, confidence interval; dLDL-C, direct low-density lipoprotein cholesterol; MAD, mean absolute deviation; MeAD, median absolute deviation.

MeADs with 95% CIs were calculated by two-sample difference. SI conversion factors: To convert cholesterol to mmol/L,

values were multiplied by 0.0259.
