# Supplementary material for: More precise method of low-density lipoprotein cholesterol estimation for tobacco and electronic cigarette smokers: A cross-sectional study
Source: PLoS One. 2024 Sep 20;19(9):e0309002. doi: 10.1371/journal.pone.0309002 (PMC11414970; doi:10.1371/journal.pone.0309002)
Supplement: S5 Table — (DOCX) [file pone.0309002.s010.docx]

S5 Table. Mean and median absolute deviations with 95% confidence intervals of estimated low-density lipoprotein cholesterol stratified by dLDL-C in the group with TG levels of <1000 mg/dL

|  | **Sampson equation** | | **Martin equation** | | **Friedewald equation** | |
| --- | --- | --- | --- | --- | --- | --- |
|  |  |  |  | Never smoker |  |  |
| **dLDL-C** | MAD | MeAD | MAD | MeAD | MAD | MeAD |
| **<40** | 8.08 | -3.34 (-8.99 to 2.31) | 8.73 | -3.41 (-8.46 to 1.64) | 19.71 | -10.91 (-23.73 to 1.91) |
| **<70** | 6.67 | -2.52 (-4.66 to -0.38) | 8.67 | -1.19 (-3.26–0.88) | 11.38 | -6.29 (-8.72 to -3.85) |
| **≥70 and <100** | 6.56 | -1.63 (-2.32 to -0.94) | 7.44 | -1.19 (-1.85 to -0.52) | 9.07 | -2.93 (-3.70 to -2.16) |
| **≥100 and <130** | 7.08 | -2.67 (-3.33 to -2.01) | 7.10 | -1.78 (-2.40 to -1.16) | 8.62 | -3.61 (-4.33 to -2.90) |
| **≥130 and <160** | 7.05 | -2.67 (-3.52 to -1.82) | 6.70 | -1.93 (-2.73 to -1.12) | 7.88 | -3.17 (-4.05 to -2.29) |
| **≥160** | 8.80 | -2.52 (-5.59 to 0.54) | 7.59 | -1.33 (-4.35 to 1.68) | 9.27 | -3.34 (-6.45 to -0.23) |
|  |  |  |  | Former smoker |  |  |
| **dLDL-C** | MAD | MeAD | MAD | MeAD | MAD | MeAD |
| **<40** | 5.27 | -1.56 (-6.21 to 3.10) | 6.91 | -6.00 (-11.16 to -0.85) | 13.88 | -10.27 (-17.44 to -3.10) |
| **<70** | 7.27 | -4.15 (-7.60 to -0.70) | 8.73 | -3.26 (-6.67–0.14) | 12.82 | -8.93 (-12.84 to -5.03) |
| **≥70 and <100** | 7.80 | -1.26 (-2.56 to 0.04) | 8.07 | -1.41 (-2.75 to -0.07) | 11.76 | -4.90 (-6.55 to -3.26) |
| **≥100 and <130** | 7.21 | -1.63 (-2.63 to -0.64) | 6.97 | -1.78 (-2.77 to -0.79) | 9.36 | -3.19 (-4.31 to -2.07) |
| **≥130 and <160** | 7.30 | -2.08 (-3.40 to -0.75) | 6.57 | -2.08 (-3.37 to -0.78) | 8.40 | -2.92 (-4.33 to -1.51) |
| **≥160** | 9.03 | -2.97 (-7.66 to 1.73) | 8.27 | -2.00 (-6.69 to 2.68) | 9.52 | -4.46 (-9.28 to 0.36) |
|  |  |  |  | Current smoker |  |  |
| **dLDL-C** | MAD | MeAD | MAD | MeAD | MAD | MeAD |
| **<40** | 8.34 | -4.15 (-9.66 to 1.36) | 8.18 | -6.08 (-11.26 to -0.90) | 19.26 | -11.09 (-21.17 to -1.01) |
| **<70** | 8.68 | -3.85 (-7.22 to -0.49) | 9.48 | -2.52 (-5.82 to 0.78) | 16.90 | -11.00 (-15.01 to -6.98) |
| **≥70 and <100** | 7.83 | -3.19 (-4.50 to -1.88) | 8.19 | -2.37 (-3.61 to -1.13) | 12.80 | -8.23 (-9.98 to -6.47) |
| **≥100 and <130** | 8.19 | -1.93 (-2.96 to -0.90) | 7.21 | -1.26 (-2.24 to -0.28) | 11.22 | -4.47 (-5.71 to -3.23) |
| **≥130 and <160** | 8.33 | -1.19 (-2.50 to 0.13) | 7.19 | -1.19 (-2.48 to 0.11) | 9.74 | -2.85 (-4.32 to -1.37) |
| **≥160** | 8.39 | -5.04 (-7.86 to -2.22) | 7.49 | -3.04 (-5.72 to -0.36) | 9.06 | -6.40 (-9.40 to -3.41) |
|  |  |  |  | Electronic cigarette smoker |  |  |
| **dLDL-C** | MAD | MeAD | MAD | MeAD | MAD | MeAD |
| **<40** |  |  |  |  |  |  |
| **<70** | 6.85 | -6.08 (-11.47 to -0.69) | 8.31 | -7.56 (-14.91 to -0.21) | 11.38 | -8.17 (-15.41 to -0.93) |
| **≥70 and <100** | 8.78 | -3.71 (-7.54 to 0.12) | 9.35 | -2.37 (-5.95 to 1.20) | 13.53 | -8.10 (-12.80 to -3.41) |
| **≥100 and <130** | 8.62 | 0.44 (-1.99 to 2.88) | 7.37 | -0.44 (-2.83 to 1.94) | 11.98 | -2.22 (-5.21 to 0.76) |
| **≥130 and <160** | 8.02 | -2.37 (-6.11 to 1.37) | 6.94 | -1.33 (-5.01 to 2.35) | 9.35 | -2.75 (-6.53 to 1.04) |
| **≥160** | 8.76 | -10.82 (-20.05 to -1.6) | 9.13 | -9.93 (-18.88 to -0.99) | 9.61 | -12.81 (-22.49 to -3.13) |

CI, confidence interval; dLDL-C, direct low-density lipoprotein cholesterol; MAD, mean absolute deviation; MeAD, median absolute deviation.

MeADs with 95% CIs were calculated by two-sample difference. SI conversion factors: To convert cholesterol to mmol/L,

values were multiplied by 0.0259
