# Supplementary material for: More precise method of low-density lipoprotein cholesterol estimation for tobacco and electronic cigarette smokers: A cross-sectional study
Source: PLoS One. 2024 Sep 20;19(9):e0309002. doi: 10.1371/journal.pone.0309002 (PMC11414970; doi:10.1371/journal.pone.0309002)
Supplement: S6 Table — (DOCX) [file pone.0309002.s011.docx]

S6 Table. Mean and median absolute deviations with 95% confidence intervals of estimated low-density lipoprotein cholesterol stratified by dLDL-C in the group with TG levels of >400 mg/dL and <1000 mg/dL

|  | **Sampson equation** | | **Martin equation** | | **Friedewald equation** | |
| --- | --- | --- | --- | --- | --- | --- |
|  | Never smoker | | | | | |
| **dLDL-C,mg/dL** | MAD | MeAD | MAD | MeAD | MAD | MeAD |
| **<40** | 19.31 | -10.38 (-31.64 to 10.88) | 18.73 | -7.56 (-26.46 to 11.34) | 25.23 | -6.45 (-32.14 to 19.24) |
| **<70** | 9.81 | -2.52 (-11.16 to 6.12) | 9.49 | -1.78 (-10.29 to 6.74) | 14.11 | -10.06 (-20.09 to -0.04) |
| **≥70 and <100** | 14.16 | -6.08 (-9.75 to -2.41) | 14.21 | -6.52 (-10.32 to -2.72) | 19.00 | -12.04 (-16.93 to -7.15) |
| **≥100 and <130** | 15.87 | -4.00 (-8.75 to 0.74) | 16.75 | -4.74 (-9.87 to 0.39) | 20.57 | -9.93 (-16.43 to -3.44) |
| **≥130 and <160** | 12.79 | -2.08 (-14.88 to 10.73) | 12.94 | -2.82 (-16.2 to 10.56) | 15.86 | -5.86 (-19.65 to 7.93) |
| **≥160** | 7.09 | -11.56 (-23.68 to 0.56) | 7.04 | -10.67 (-22.47 to 1.12) | 8.49 | -15.12 (-28.67 to -1.57) |
|  | Former smoker | | | | | |
|  | MAD | MeAD | MAD | MeAD | MAD | MeAD |
| **<40** | 2.38 | -1.78 (-7.88 to 4.33) | 4.38 | -2.22 (-12.21 to 7.76) | 8.87 | -8.03 (-29.02 to 12.96) |
| **<70** | 9.91 | -5.34 (-9.82 to -0.85) | 10.36 | -7.12 (-11.96 to -2.27) | 16.99 | -15.21 (-21.71 to -8.72) |
| **≥70 and <100** | 13.94 | -3.85 (-8.20 to 0.49) | 14.28 | -4.74 (-9.28 to -0.21) | 20.09 | -10.97 (-17.15 to -4.79) |
| **≥100 and <130** | 13.92 | -5.04 (-9.64 to -0.44) | 13.90 | -4.45 (-9.12 to 0.22) | 18.49 | -7.39 (-12.93 to -1.84) |
| **≥130 and <160** | 9.53 | -3.26 (-10.95 to 4.42) | 9.04 | -5.63 (-13.73 to 2.47) | 12.09 | -6.95 (-15.99 to 2.09) |
| **≥160** | 9.01 | -8.15 (-40.95 to 24.64) | 9.38 | -13.79 (-48.65 to 21.07) | 11.27 | -11.83 (-46.48 to 22.83) |
|  | Current smoker | | | | | |
|  | MAD | MeAD | MAD | MeAD | MAD | MeAD |
| **<40** | 10.91 | -4.45 (-16.72 to 7.83) | 10.83 | -5.04 (-17.54 to 7.46) | 22.76 | -24.53 (-52.80 to 3.73) |
| **<70** | 11.87 | -9.34 (-13.88 to -4.80) | 12.45 | -7.56 (-11.66 to -3.47) | 20.23 | -17.49 (-23.57 to -11.42) |
| **≥70 and <100** | 12.39 | -6.38 (-9.44 to -3.31) | 12.85 | -5.93 (-8.99 to -2.87) | 18.39 | -10.45 (-14.28 to -6.62) |
| **≥100 and <130** | 11.38 | -3.41 (-6.81 to -0.01) | 11.85 | -3.26 (-6.73 to 0.21) | 16.05 | -8.99 (-13.62 to -4.36) |
| **≥130 and <160** | 11.06 | -6.89 (-11.88 to -1.91) | 11.01 | -6.60 (-11.57 to -1.63) | 14.36 | -10.95 (-17.22 to -4.68) |
| **≥160** | 10.19 | -3.71 (-15.09 to 7.67) | 12.17 | -11.12 (-26.19 to 3.96) | 12.40 | -7.71 (-21.00 to 5.58) |
|  | Electronic smoker | | | | | |
|  | MAD | MeAD | MAD | MeAD | MAD | MeAD |
| **<40** |  |  |  |  |  |  |
| **<70** | 12.83 | -15.12 (-39.4 to 9.16) | 13.00 | -13.71 (-37.40 to 9.97) | 17.05 | -19.45 (-50.38 to 11.47) |
| **≥70 and <100** | 12.49 | -9.41 (-21.75 to 2.92) | 13.72 | -7.49 (-20.35 to 5.37) | 18.32 | -12.07 (-26.02 to 1.87) |
| **≥100 and <130** | 11.33 | -3.71 (-11.26 to 3.84) | 11.56 | -6.67 (-13.68 to 0.33) | 15.65 | -10.38 (-20.1 to -0.65) |
| **≥130 and <160** | 6.60 | 3.11 (-11.47 to 17.7) | 5.80 | 4.89 (-3.52 to 13.31) | 7.02 | 3.47 (-9.08 to 16.02) |
| **≥160** | 15.95 | -2.89 (-62.34 to 56.56) | 15.35 | -2.00 (-59.01 to 55.01) | 17.48 | -5.15 (-71.07 to 60.76) |

CI, confidence interval; dLDL-C, direct low-density lipoprotein cholesterol; MAD, mean absolute deviation; MeAD, median absolute deviation.

MeADs with 95% CIs were calculated by two-sample difference. SI conversion factors: To convert cholesterol to mmol/L,

values were multiplied by 0.0259
